# Determination of Metal Content in *Crocus sativus *L. Corms in Dormancy and Waking Stages

**Published:** 2013

**Authors:** Nardana Esmaeili, Hassan Ebrahimzadeh, Khosrou Abdi, Masoud Mirmasoumi, Navid Lamei, Mehrdad Azizi Shamami

**Affiliations:** a *Department of Plant Biology, School of Biology, College of Sciences, Tehran University, Tehran, Iran.*; b *Department of Medicinal Chemistry and Radiopharmacy, Faculty of Pharmacy, Tehran University of Medical Sciences, Tehran, 14174, Iran .*; c *Drug Design and Development Research Center, Tehran University of Medical Sciences, Tehran, Iran.*; d *Nuclear Science and Technology Research Institute-Atomic Energy Organization, Tehran, Iran.*

**Keywords:** *Crocus sativus *L., Corms, Mineral elements, Inductively Coupled Plasma-Optical Emission Spectroscopy, Neutron Activation Analysis, Atomic Absorption Spectrophotometry

## Abstract

More than 30 mineral elements have been found with different key functions in helping plants and animals to survive and live healthy. As a direct result, they have always attracted the attention of scientists. The quest is to find some efficient analytical and quantitative procedures in this study to determine some mineral and trace elements of Iranian *Crocus sativus *L. corms. Several studies have been made using distinct methods and eventually, to achieve this purpose, three analytical methods were used as follows: Neutron Activation Analysis (NAA), Inductively Coupled Plasma-Optical Emission Spectroscopy (ICP-OES) and Atomic Absorption Spectrophotometry (AAS). Seventeen mineral and trace elements (Mg, Na, Ca, K, Mn, Zn, Cu, Pb, Hg, Ni, Fe, Co, Cd, Sr, Rb, Sc, and Br) were determined in *Crocus sativus *L. corms in two different physiological stages.

The mineral elements content in saffron corms showed a wide variability and their concentrations in dormancy stage were higher than waking. Despite of the fact that K concentration was the highest among all mineral elements studied in both samples, it was nil for Sc, Co, Hg, Pb and Cd.

## Introduction

 Plants have always attracted the attention of scientists, especially nowadays, as our green planet is less green every day (1). *Crocus sativus* L., commonly known as saffron, is a perennial stemless herb of the *Iridaceae *family, widely cultivated in Iran and other countries, such as Spain and Greece (2-4). *Crocus sativus *is cultivated since ancient times as a source of saffron (5). *Crocus sativus *L. is a triploid plant and sterile geophyte propagated by replacement corms in period of dormancy (6). The past studies mainly focused on its Saffron’s pharmacology and clinical application. However, its major metal elements have not been studied adequately and only two investigations examined the contents of seven or eight major metal elements in Saffron (7).

 Many elements, in trace amounts, play a vital role in metabolic processes and are essential for the general well being of humans (8). These elements are contained in enzymes and activate them, thereby in an essential way influencing biochemical processes in cells (9). Several elements, such as Na, K, Mg and Mn are present at mg g-1 level, whereas elements such as Cr, Fe, Co, Ni, Cu, Zn and Cd are present at a few μg g^-1^. In addition, some rare elements have been reported at the ng g^-1^ level (10).

 The content of essential elements in plants seems to be conditional, being affected by the geochemical characteristics of the soil and by the ability of plants to selectively accumulate some of these elements (11). Bioavailability of the elements depends on the form of their bond with the constituents of a soil. Plants readily assimilate such compounds through the roots which are dissolved in waters and occur in ionic forms. Additional sources of these elements for plants include rainfall, atmospheric dusts, plant protection agents and fertilizers, which could be adsorbed through the leaf blades (9). Many medicinal herbs and their compounds can present a health risk due to the presence of toxic elements such as Pb, Cd, Al, Hg and other elements like Cr, which are hazardous to humans, depending on their oxidation states and present at high concentrations (12).

 It is well known that some elements, depending on their concentrations, can play different roles in plant life. Zinc, Mn and Fe are important co-enzymes; Cu is bound to amino acids while Mn and Ce bind to some bio-macromolecules forming coordination compounds (8). Hence, better understanding of the uptake mechanism of elements, their functions in plant metabolism and their possible toxic effect are of great importance in both basic and applied plant studies. In that course, an accurate quantitative analysis at sensitivities down to trace and ultra-trace levels should always be made. Little has been published on the chemical composition or biochemistry of *C. sativus *corms (2). As a contribution in that sense and due to the importance of the mineral elements in the corms of *Crocus sativus*, and also its usage as a food resource in some parts of Iran, this work is dedicated to the determination of the total analytical contents of some major, minor and trace elements in *Crocus sativus *L. corms in two different physiological stages, cultivated in Iran. Three different analytical methods, inductively coupled plasma-optical emission spectroscopy, neutron activation analysis (13) and flame atomic absorption spectrometry (1) were applied in this research.

## Experimental

*Chemicals*

 Standard solutions (1000 μg mL^-1^) of each element used by atomic absorption and ICP-OES methods were purchased from Merck. Deionized water (Millipore, Darmstadt, Germany) was used throughout this study for serial dilution of standards. All solvents and reagents such as H_2_O_2_ and sulfuric acid were of analytical reagent grade (Merck).

*Apparatus*

 A Hack country of USA of Digestive apparatus was used for digestion of samples. The determination of Ca, Fe, Zn, Mn, Ni, Cu, K, Co, Pb, Hg, Cd, Mg and Na was performed on a model Vista MPX Varian inductively coupled plasma-optical emission spectroscopy (ICP-OES) under optimized measurement condition. Instrument configuration and general experimental conditions for ICP-OES are given in Table 1.

**Table 1:** ICP-OES operating conditions for determination of some elements in *Crocus sativus *corms

**Rf power (KW)**	1.3
**Plasma argon flow rate (L/min)**	15
**Auxiliary argon flow rate (L/min)**	1.5
**Nebulizer press (KPa)**	150
**Sample uptake (S)**	20
**Elements monitored (Wavelength, nm)**	Cu (324.754), Fe (259.94), Zn (213, 857), Mn (257.61), Mg (279.553), K (766.491), Na ( 589.592), Ca (396.847), Hg (184.887), Pb ( 220.353), Cd (214.439), Co (238.892), Mo (202.032), Ni (232.057)

 The other following apparatuses were used for this research: A Varian AA 220 atomic absorption spectrophotometer was used for the analysis of Mg, Mn, Ca, Zn, Fe, K, Na and a Varian 110 GTA graphite furnace was performed for Cu, Ni, Co, Hg, Pb and Cd analysis. The elements were measured by the optimum operating conditions with an air-acetylene flame.

Tehran Research Reactor (TRR) (UNITED STATE OF AMERICA) with maximum Thermal power (5MW) and P-type HPGE (High Pure Germanium) Semiconductor Detector (CANBERRA Industries, Inc. Detector Model: 7229N, Crystal Model: 7500SL and Operating Voltage: + 2500 VOLTS) were used for the analysis by NAA (Neutron Activation Analysis).

*Sample preparation*

*Crocus sativus *L corms were collected from the Tehran University farm, located in Karaj, near Tehran. Dormant and Waking corms were collected during August and March, respectively. These corms depleted from their sheeting leaves and cleaned from any dirt particles. Then were washed and dried for 24 h under 60°C ovens.

For being analyzed by atomic absorption and ICP-OES, 1 g of each dried sample was accurately weighed and taken in a 100 mL beaker of digestion apparatus, to which 5 mL of sulfuric acid was added and heated up to slurry formation. On further heating, a white gelatinous mass was observed and then, 15 mL of H_2_O_2_ was added and the contents were reduced to 2 mL by evaporation. Next, the solution was made up to 25 mL by adding deionized water. All the solutions were stored in tightly capped glass bottles.

For the analysis by NAA, closely weighed samples (30-50 mg) of dry mass of saffron corms powder were packed in high-density polythene bags. Elemental standards were prepared by depositing 30 mg of IAEA-V-10, tracing the element in Hay (powder), to an Al foil and packed. These samples, along with elemental standard, were irradiated in Rabbit Beam Tube for 1 h at a thermal neutron flux of 10^10^ n cm^-2^ s^-1^ in the 5MW Tehran Research Reactor center, Tehran. After appropriate cooling time, *γ*-activities of activation products were measured by high resolution gamma ray spectrometry, using p-type-HPGE detector. The samples were counted 3 and 24 days after the irradiation, triplicate. The software for data collection and analysis were Mastro 4 and SPAN, respectively.

*Statistical analysis*

Statistical analysis was performed using SPSS 11, one-way ANOVA followed by repeated measurement of multiple comparisons. The p-values less than 0.05 were considered statistically significant.

## Results and Discussion

In the present work, seventeen elements were determined in *Crocus sativus *corms in two physiological stages by three different methods and their concentrations were compared with each other. In Tables 2 and 3, the mean values of calculated concentrations for waking and dormant corms are presented, respectively. Standard deviation was calculated on the basis of triplicate measurements or counting statistics. On the other hand, the relative standard deviations (RSDs) in the most cases were found < 10% suggesting precision of our measurements.

At first glance, it can be indicated that the results present a wide variation. As shown in Table 2, for all determined elements by NAA method, the highest concentration was found for K, namely (4870 ± 127.6 μg g^-1^) and the lowest content was for Sc (0.001 ± 0.008 μg g^-1^). Moreover, Table 3 shows the same results for dormant corms, (6673 ± 253.3 μg g^-1^) and (0.001 ± 0.008 μg g^-1^) for K and Sc, respectively.

**Table 2:** Metal content (μg/g) of *Crocus sativus *waking corms analyzed by three different methods. Results are presented as mean ± SD (n = 3); ND: Not Detected. *: analyzed with graphite furnace.

**ICP-OES Concentration (μg/g) RSD%**			**Neutron Activation Concentration(μg/g) RSD%**		**Atomic Absorption Concentration(μg/g) RSD%**	
Mg	692.9 ± 2.7	0.39	665.8 ± 35.4	5.3	680.9 ± 27.9	4
Mn	4.8 ± 0.4	8.8	6.1 ± 0.4	6.5	5.06 ± 0.3	6
Ca	2868.3 ±121.7	4.2	3110.3 ± 27.7	0.89	2980.8 ± 111.5	3.7
Cu	1.4 ± 0.14	10	1.03 ± 0.12	11.6	1.33 ± 0.04 ^*^	2.9
Zn	18.23 ± 0.77	4.26	16.2 ± 0.37	2.3	18.3 ± 0.6	3.6
Fe	10.92 ± 0.8	7.3	11.2 ± 0.4	2.6	10.65 ± 0.6	5.7
K	4492 ± 142.4	3.1	4870 ± 127.6	2.6	4395.6 ± 60.1	1.4
Na	50.6 ± 2.1	4.1	48.3 ± 1.4	2.9	52.46 ± 1.15	2.2
Ni	3.23 ± 0.2	6.4	2.9 ± 0.3	10	2.59 ± 0.4 ^*^	9.2
Br	----------	------	0.13 ± 0.006	4.3	----------	------
Sc	----------	------	0.001 ± 0.0002	7.9	----------	------
Co	0.02 ± 0.002	11.8	0.02 ± 0.001	7.3	0.03 ± 0.002^ *^	8.5
Hg	ND	------	0.003 ± 0.0002	7.4	ND ^*^	------
Pb	0.010 ± 0.002	20	0.009 ± 0.0003	3.3	0.009 ± 0.001^ *^	11.1
Cd	0.009 ± 0.001	11.1	0.008 ± 0.0006	7.5	0.007 ± 0.001^*^	14.2
Rb	----------	------	2.48 ± 0.16	6.4	----------	------
Sr	----------	------	ND	ND	----------	------

In a parallel approach, atomic absorption was the other method of choice we used for quantification of mineral elements. A glimpse at the Tables (Tables 2 and 3) reveals that as an overall trend, the concentrations of all elements in dormant corms analyzed by this method underwent a rising trend with the exception of Zn content in comparison with waking sample. This results show that, K has the highest concentration (4395.6 ± 60.1 μg g^-1^) in waking corms and (6512 ± 31.37 μg g^-1^) in dormant (Tables 2 and 3). As mentioned above, there is an increase in Cu concentration in dormant corms compared with waking, by approximately 2 to 1, which may be because of the higher activity of polyphenol oxidase enzyme in that stage.

**Table 3:** Metal content (μg/g) of *Crocus sativus *dormant corms analyzed by three different methods. Results are presented as mean ± SD (n = 3); ND: Not Detected. *: analyzed with graphite furnace.

**ICP-OES Concentration (μg/g) RSD%**			**Neutron Activation Concentration(μg/g) RSD%**		**Atomic Absorption Concentration(μg/g) RSD%%**	
Mg	830 ± 4.5	0.55	826.6 ± 3.05	0.37	838.5 ± 17.5	2.1
Mn	5.7 ± 0.2	5.4	5.9 ± 0.1	1.7	6.02 ± 0.5	7.8
Ca	2833 ± 56.9	2	2994 ± 14.5	0.48	3079 ± 94.06	3
Cu	3.18 ± 0.08	2.5	3.4 ± 0.3	8.8	2.9 ± 0.2^*^	6.9
Zn	11.3 ± 0.06	0.58	12.7 ± 0.32	2.5	11.42 ± 0.63	5.5
Fe	36 ± 0.3	0.83	38.3 ± 1.17	3.06	36.6 ± 2.16	5.9
K	6485 ± 6.5	0.1	6673 ± 253.3	3.8	6512 ± 31.37	0.48
Na	113.3 ± 5.9	5.2	107.8 ± 2.6	2.4	116.35 ± 2.9	2.5
Ni	ND	ND	4.43 ± 0.4	9.1	3.96 ± 0.4^*^	10
Br	----------	------	0.24 ± 0.01	4.4	----------	------
Sc	----------	------	0.001 ± 0.0003	8.7	----------	------
Co	0.024 ± 0.003	14.4	0.02 ± 0.002	8.1	0.033 ± 0.002^*^	7.7
Hg	ND	------	0.01 ± 0.001	9.2	ND*	------
Pb	0.013 ± 0.003	24.1	0.009 ± 0.001	11.1	0.009 ± 0.0006^*^	7.2
Cd	0.007 ± 0.001	14.2	0.009 ± 0.0008	8.8	0.008 ± 0.001^*^	12.5
Rb	----------	------	1.74 ± 0.06	3.7	----------	------
Sr	----------	------	4.4 ± 0.26	5.9	----------	------

 The ICP-OES analysis results demonstrate the high concentration of K in both samples, (6485± 6.5 μg g^-1^) and (4492 ± 142.4 μg g^-1^) for dormant and waking corms, respectively. However, the results obtained in this research are in consistent with other experiments carried on stigma, showing that Saffron contained many major metal elements (Ca, Fe, Mg, Mn, Zn, Co, Ni), which are necessary for human health (5). Results also show that, the content of K obtained by three different methods as said above was the highest content among other elements and varied between 4395 and 6673 μg g^-1^, according to the physiological stage and analytical methods. In addition, low contents of heavy metals (Cu, Pb, Hg, Cd) are also important for the quality of plant.

It is known that many organic and inorganic materials can be dissolved during the digestion process. Therefore, a direct method of NAA is the most suitable way for determination of elements to eliminate the matrix interferences compared to AAS and ICP-OES methods (6).

Uptake of various elements by plants through the root system from the soil depends on particular plant, botanical structure of specific tissue, soil type and element as well. Besides, microelements can enter the plant from the external environmental compartments. On the other hand, essential elements content in living organisms depends on physiological processes, being a part of regulatory mechanisms which are able to keep the elements below toxic level. In this investigation, the higher content of mineral elements in dormancy stage compared with waking may be due to the immigration of these elements from every possible routes of this plant to corms because of the storage role of corms in summer till next waking stage. The abundance of K, Mg and Ca in this analysis was in agreement with previous findings that these three metals represent the most abundant metal constituents of many plants (14). Statistical analysis was carried out to determine the significance of various results obtained with different methods showed no significant differences between three analytical applied methods. There is a significant difference (p < 0.01) in the concentrations of Br and Rb in two different physiological stages and their concentrations were higher and lower in dormancy compared with waking, respectively. Furthermore, results showed a significant difference (p < 0.05) in the contents of Cu, Zn, Fe, Na, Ni as their concentrations were higher in dormancy than waking stages with the exception of Zn. Interestingly, no significant difference was seen in the concentrations of Mg, Mn, Ca, K, SC, Co, Hg, Pb and Cd.

## Conclusion

Three different methods were established for the determination of some mineral elements in Medicinal Iranian Saffron Corms. Wide ranges of varieties were seen in the amount of mineral elements in corms analyzed by different methods which may suggest that the concentration levels depend on, especially, the environmental conditions and the physiological states of plants. Although the *Crocus sativus *corms are rich resources of Ca and K, some other major elements like Fe, Mg, Mn, Zn, Co and Ni were also detected which are necessary for human health. In addition to the above major elements, the contents of heavy metals were also determined. Their concentrations were low and all meet the national hygiene standards for safely being used by humans.
